# Hypoxia Routes Tryptophan Homeostasis Towards Increased Tryptamine Production

**DOI:** 10.3389/fimmu.2021.590532

**Published:** 2021-02-19

**Authors:** Soumya R. Mohapatra, Ahmed Sadik, Suraj Sharma, Gernot Poschet, Hagen M. Gegner, Tobias V. Lanz, Philippe Lucarelli, Ursula Klingmüller, Michael Platten, Ines Heiland, Christiane A. Opitz

**Affiliations:** ^1^ DKTK Brain Cancer Metabolism Group, German Cancer Research Center (DKFZ), Heidelberg, Germany; ^2^ Department of Arctic and Marine Biology, UiT The Arctic University of Norway, Tromsø, Norway; ^3^ Centre for Organismal Studies (COS), University of Heidelberg, Heidelberg, Germany; ^4^ DKTK Clinical Cooperation Unit Neuroimmunology and Brain Tumor Immunology, German Cancer Research Center (DKFZ), Heidelberg, Germany; ^5^ Department of Neurology, Medical Faculty Mannheim, Mannheim Center for Translational Neurosciences (MCTN), Heidelberg University, Mannheim, Germany; ^6^ Division of Immunology and Rheumatology, Department of Medicine, Stanford University School of Medicine, Stanford, CA, United States; ^7^ Division Systems Biology of Signal Transduction, German Cancer Research Center (DKFZ), Heidelberg, Germany; ^8^ Translational Lung Research Center (TLRC), Member of the German Center for Lung Research (DZL), Heidelberg, Germany; ^9^ Neurology Clinic and National Center for Tumor Diseases, University Hospital of Heidelberg, Heidelberg, Germany

**Keywords:** liver, hypoxia, tryptophan, TDO2, regulation, AHR activity, hallucination, tryptamine

## Abstract

The liver is the central hub for processing and maintaining homeostatic levels of dietary nutrients especially essential amino acids such as tryptophan (Trp). Trp is required not only to sustain protein synthesis but also as a precursor for the production of NAD, neurotransmitters and immunosuppressive metabolites. In light of these roles of Trp and its metabolic products, maintaining homeostatic levels of Trp is essential for health and well-being. The liver regulates global Trp supply by the immunosuppressive enzyme tryptophan-2,3-dioxygenase (TDO2), which degrades Trp down the kynurenine pathway (KP). In the current study, we show that isolated primary hepatocytes when exposed to hypoxic environments, extensively rewire their Trp metabolism by reducing constitutive *Tdo2* expression and differentially regulating other Trp pathway enzymes and transporters. Mathematical modelling of Trp metabolism in liver cells under hypoxia predicted decreased flux through the KP while metabolic flux through the tryptamine branch significantly increased. In line, the model also revealed an increased accumulation of tryptamines under hypoxia, at the expense of kynurenines. Metabolic measurements in hypoxic hepatocytes confirmed the predicted reduction in KP metabolites as well as accumulation of tryptamine. *Tdo2* expression in cultured primary hepatocytes was reduced upon hypoxia inducible factor (HIF) stabilisation by dimethyloxalylglycine (DMOG), demonstrating that HIFs are involved in the hypoxic downregulation of hepatic *Tdo2*. DMOG abrogated hepatic luciferase signals in *Tdo2* reporter mice, indicating that HIF stability also recapitulates hypoxic rewiring of Trp metabolism *in vivo*. Also in WT mice HIF stabilization drove homeostatic Trp metabolism away from the KP towards enhanced tryptamine production, leading to enhanced levels of tryptamine in liver, serum and brain. As tryptamines are the most potent hallucinogens known, the observed upregulation of tryptamine in response to hypoxic exposure of hepatocytes may be involved in the generation of hallucinations occurring at high altitude. KP metabolites are known to activate the aryl hydrocarbon receptor (AHR). The AHR-activating properties of tryptamines may explain why immunosuppressive AHR activity is maintained under hypoxia despite downregulation of the KP. In summary our results identify hypoxia as an important factor controlling Trp metabolism in the liver with possible implications for immunosuppressive AHR activation and mental disturbances.

## Introduction

The liver and its role in the human physiology has intrigued people for centuries (1). With advances in modern medicine and a better understanding of human physiology, the liver has been revealed as the central gatekeeper for all metabolic inputs, being responsible for systemic metabolic homeostasis (1, 2). This master regulator of metabolic inputs maintains steady state conditions during times of plenty by metabolizing and channeling excessive nutrients for storage, while during starved conditions the previously stored nutrients are released for use by peripheral tissues (2, 3). A class of nutrients that are acquired exclusively through dietary sources are essential amino acids, for example Trp. Dietary Trp upon absorption in the gut exists in the blood with a majority bound to albumin while a minor fraction (5–10%) exists as free Trp, with the existence of a rapid equilibrium between the two (4). The liver regulates systemic free Trp levels by degrading Trp into various metabolic products. Trp can be degraded along three known minor metabolic pathways namely serotonin, tryptamine and indolepyruvic acid pathways, however the liver degrades a majority of dietary Trp through the KP (5, 6). To accomplish this, hepatocytes endogenously express TDO2, which catalyses the first step of the KP (7, 8).

In metabolically active tissues such as liver, oxygen availability is an important factor not only for cell survival but also for maintaining metabolic function as well as energy metabolism of the cell (9). In the liver, cellular differentiation is also regulated by oxygen availability both during liver development as well as liver regeneration (10, 11). Cells exposed to hypoxic conditions generally modulate the expression of a wide range of genes through the upregulation of HIFs (12). Under normoxic conditions, HIF proteins are degraded by prolyl-hydroxylase (PHD) enzymes, however onset of hypoxia inhibits the action of these enzymes thus stabilizing the HIF transcription factors, the most prominent among which is HIF1α (13). Upon stabilization, HIF1α translocates into the nucleus and modulates (i.e. both up- or down-regulates) the expression of hypoxia target genes (9, 10). Stabilization of HIF1α has also been reported in various hepatic diseases such as hepatic ischemia reperfusion (IR) injury (14, 15), alcohol-mediated liver injury (16), liver fibrosis (17), viral hepatitis (18) as well as in hepatocellular carcinoma (19, 20).

We previously found that HIF1α downregulates the expression of the Trp-catabolizing enzyme tryptophan-2,3-dioxygenase (TDO2) in human glioblastoma cells (21). However, it is known that the regulation of TDO2 differs between glioblastoma cells and liver cells, indicating that TDO2 may be regulated in a tissue-specific manner (22). Given the prevalence and importance of hypoxia in hepatic development, as well as liver pathology, we therefore set out to decipher the effects of hypoxia on Trp catabolism in the liver. We report that hypoxia significantly alters the homeostatic Trp catabolism in the liver by favouring the production of tryptamine and its downstream metabolites, instead of the usual KP metabolites.

## Materials and Methods

### Isolation of Primary Murine Hepatocytes

Isolation of primary hepatocytes from mice was approved under the reference number A24/10 and G14/17 by the governmental review committee on animal care of Baden-Württemberg, Germany. C57BL/6N mice were obtained from Charles River and housed at the DKFZ animal facility under a constant day light cycle. Animals were allowed ad libitum access to a standard mouse diet and water. Murine hepatocytes were harvested from male animals aged between 8 to 10 weeks according to a standardized protocol described earlier (23). Harvested hepatocytes were seeded at a density of 1x10^6^ cells/well, in 6 well collagen I-coated tissue culture plates (BD Biosciences), in 2 mL of adhesion medium and allowed to attach for 4 h at normoxic conditions i.e. 18.6% O_2_ concentration (24) in a SANYO MCO-18AIC incubator with 5% CO_2_ and at 37°C. The adhesion medium consisted of phenol red-free Williams E medium (Biochrom) containing 10% (v/v) fetal bovine serum (Life Technologies), 0.1 µM dexamethasone, 10 µg/mL insulin, 2 mM L-glutamine and 1% (v/v) 100X penicillin/streptomycin (Life Technologies).

Following the 4 h of incubation, wells were washed twice with PBS (Gibco) to remove unattached hepatocytes. This was followed by an overnight incubation of the cells in serum-free medium. The serum-free medium was composed of phenol red-free Williams E medium containing 0.1 µM dexamethasone, 2 mM L-glutamine, and 1% (v/v) 100X penicillin/streptomycin. Following the overnight incubation, the serum-free medium was removed, cells were again washed three times and 2 ml of serum and dexamethasone-free medium was added for initiation of the hypoxia or hypoxia mimetic treatments according to the respective experimental conditions described in subsequent sections.

### Hepatocytes Exposure to Low Oxygen Conditions

Murine hepatocytes cultured in 6 well plates in serum and dexamethasone-free medium were subjected to either normoxic or hypoxic conditions for 1, 2 and 3 days. A Labotect incubator C42 was used to attain hypoxic conditions i.e. 1% O_2_ concentration with 5% CO_2_ and 37°C. Cells were always seeded in duplicate for each time point with the control plate being incubated in the normoxic incubator while the second was incubated in the hypoxia incubator. Cells were lysed for RNA extraction at the end of the respective time points.

### Treatment of Hepatocytes With a Chemical HIF Protein Stabilizing Agent

The well known chemical mimetic of hypoxia i.e. dimethyloxalylglycine (DMOG) was used for HIF protein stabilization. 20M stocks of DMOG (Frontier Scientific Inc.) were prepared in ethanol (Sigma). Hepatocytes were treated such that they were exposed to 3 mM of DMOG in a well as final treatment condition.

### RNA Isolation and Quantitative (q)RT-PCR

Total RNA was isolated from the harvested samples using a Qiagen RNAeasy Mini Kit (Qiagen). Subsequently, a High Capacity cDNA reverse transcriptase kit (Applied Biosystems) was used for cDNA production from 1 µg total RNA. (q)RT-PCR was performed using the SYBR Select Master Mix (Thermo Fisher Scientific) on a StepOnePlus real-time PCR system (Applied Biosystems). For all (q)RT-PCR measurements *18s Rna* was used as a housekeeping gene for normalization during analysis. Unless stated otherwise, all the murine (q)RT-PCR primers for a target gene used in the present study were designed using Primer Blast (NCBI). The murine *Ndrg1* primers used in the study were taken from a prior publication by Okuda and colleagues (25). The murine primer sequences used are listed below:

**Table:** 

*Tdo2*	Forward	5’- GAATGCGCAAGAACTTCAG -3’
	Reverse	5’- TTCCAGAACCGAGAACTGCT -3’
*18s Rna*	Forward	5’- GCAATTATTCCCCATGAACG -3’
	Reverse	5’- GGCCTCACTAAACCATCCAA -3’
*Ndrg1*	Forward	5’- ACCCTGAGATGGTAGAGGGTCTC -3’
	Reverse	5’- CCAATTTAGAATTGCATTCCACC -3’
*Ddc*	Forward	5’- AGTAAGTTGCCCAAGCACCA -3’
	Reverse	5’- ACGGGAATCCATGTTGAAAATTG -3’
*Inmt*	Forward	5’- CTGGAGGGAGACAGAAGCAG -3’
	Reverse	5’- ACACTCCATGGCCAGAAAGG -3’
*Kyat1*	Forward	5’- CCCCTGGGTGGAGTTTACCA -3’
	Reverse	5’- GTGGAGGGTAACCAAACGCT -3’
*Kynu*	Forward	5’- AAAAAGAGGAGTCGTTTGTGAC -3’
	Reverse	5’- TGAAAACTCCATCACCTTTCAGTG-3’
*Areg*	Forward	5’- GCTGAGGACAATGCAGGGTAA -3’
	Reverse	5’- AGTGACAACTGGGCATCTGG-3’
*Tiparp*	Forward	5’- TTGGAAATTCTTCTGTAGAGACCAC -3’
	Reverse	5’- CTTCTTCAATTAGTCGAACAACAGAC -3’

### Metabolic Analysis of Trp and Its Metabolites in Cultured Hepatocytes by HPLC

For metabolic analysis, 1 ml cell culture supernatant from each treatment was harvested and was snap frozen in liquid nitrogen. Subsequently, the cells were harvested by trypsinization in 1.5 ml PBS for intracellular metabolic measurements. Ten microliter of cell suspension were used for cell counting, while the rest were pelleted for snap freezing in liquid nitrogen. The separated 10 µl of cell suspension was mixed in a 1:1 ratio with Trypan Blue dye (Gibco) and subsequently the cell count was measured using an automated Cell Counter (Countess, Invitrogen). Intracellular metabolite extraction was performed with 0.1 ml 6% perchloric acid per million cells in an ultrasonic ice-bath for 10 min. Supernatants were mixed in a 1:1 ratio with 12% perchloric acid and incubated on ice for 10 min. Subsequently samples were centrifuged at maximum speed and 4°C to remove remaining cell debris. Separation was achieved on an Acquity H-class UPLC system (Waters) using an Acquity HSS T3 column (100 × 2.1 mm, 1.7 µm, Waters), which was maintained at 37°C. Clear separation of Trp and Trp downstream metabolites was achieved by increasing the concentration of solvent B (Acetonitrile) in solvent A (20 mM sodium acetate, 3 mM zinc acetate, pH 6). An Acquity FLR detector (Waters) was used for detecting Trp, 3-hydroxyanthranilic acid (3HAA), kynurenic acid (KynA) and tryptamine by fluorescence (excitation: 254 nm, emission: 401 nm). While for kynurenine (Kyn) and 3-hydroxy-kynurenine (3HKyn) an Acquity PDA detector (Waters) was used for measuring absorption at 365 nm. UPLC grade standards (Sigma) were used for quantification of all analytes. The Empower3 software suite (Waters) was used for data acquisition and processing.

### DMOG Treatment for Metabolic Measurements in WT C57BL/6 Mice

Ten week old male WT C57BL/6 mice were obtained from Charles River and housed at the DKFZ under a constant day light cycle. Three mice each were injected i.p. either with saline control (0.9% NaCl) (Fresenius Kabi) or with 8 mg/mouse DMOG (Echelon Biosciences) dissolved in saline (0.9% NaCl) (Fresenius Kabi). Twenty-four hours post treatment, the animals were sacrificed for collection of blood serum and tissue samples from liver and brain for metabolic analysis of the Trp metabolic products kynurenine and tryptamine. The experiments were approved under the animal grant number G240/18 by the governmental review committee on animal care of Baden-Württemberg, Germany.

### LC-MS/MS Based Metabolic Analysis of Trp and Its Metabolites in Frozen Tissue and Serum

Frozen liver and brain tissue was processed following an adjusted extraction protocol targeting tryptophan and kynurenine metabolites (26, 27). Briefly, samples were crushed using a ball mill (MM400, Retsch) with liquid nitrogen cooled beakers and stainless-steel balls for 1 min (brain) and 1.50 min (liver) at its highest frequency (30 Hz). The samples were then weighed for later normalization and subsequently mixed with 100 µl acidified mobile phase (0.2% formic acid + 1% acetonitrile in H2O) and 400 µl ice cold methanol.

Similarly, 15 µl of plasma was mixed with 35 µl of acidified mobile phase and 200 µl of methanol. Afterwards, all samples were kept at −20°C for 30 min to precipitate all protein. Subsequently, the samples were centrifuged for 15 min at 4°C at 16.400 g and the resulting supernatant was transferred to a new 1.5 ml microcentrifuge tube (Eppendorf). Finally, the supernatant was dried using the Eppendorf Concentrator Plus set to no heat and resuspended in 40 µl acidified mobile phase.

For metabolite separation and detection, an Acquity I-class Plus UPLC system (Waters) coupled to an QTRAP 6500+ (SCIEX) mass spectrometer with electrospray ionization (ESI) source was used. In detail, metabolites were separated by reversed phase chromatography on an Acquity HSS T3 column (150 mm x 2.1 mm, 1.7 µm, Waters) kept at 20°C and a flow rate of 0.4 ml/min. An overview of multiple reaction monitoring (MRM) transitions that were used can be found in **Table S1**. Clear separation of tryptophan and tryptophan-derived compounds was achieved by increasing the concentration of solvent B (Acetonitrile + 0,1% formic acid) in solvent A (H2O + 0.1% formic acid) as follows: 1 min 5% B, 11 min 40% B, 13 min 95% B, 15 min 95% B, and return to 5% B in 5 min. Data acquisition and processing was performed with the Sciex OS software suite (SCIEX).

### Microarray Analysis

One hundred nanogram total RNA was used for generating labeled ss-cDNA using an Affymetrix WT PLUS Reagent Kit. The Affymetrix Mouse Gene 2.0 ST chip was used for hybridization of 5.5 µg fragmented and labeled ss-cDNA for 17 h at 45°C. In accordance to the GeneChip R Expression Wash, Stain and Scan Manual for Cartridge Arrays (P/N702731) an Affymetrix GeneChip R Scanner 3000 was used for scanning the hybridized chips. The Raw CEL files were imported from disk followed by RMA normalization and summarization using the *oligo* package and were annotated at the probeset level using NetAffx (28). Differential gene expression was conducted by fitting linear model and estimating a moderated t-statistic followed by eBayes adjustment as described in the *limma* package (29, 30). All analyses were run in R, version 3.4.4, (https://cran.r-project.org/) and Bioconductor version 3.6 (https://bioconductor.org/). All graphical representations were generated using *ggplot2*, *ggpubr*, and *RcolorBrewer*. All datasets generated during the current study, have been made publicly available in the Gene Expression Omnibus (GEO) repository under accession number GSE159320.

### Mathematical Modelling of Trp Metabolism

To simulate Trp metabolism in murine hepatocytes, we used the mathematical model of Trp metabolism previously published by Stavrum et al. (31). Expression data generated for murine hepatocytes exposed to normoxia or hypoxia for 48 h were integrated into the model as described in Schäuble et al. (32) and COPASI 4.28 (33) was used to calculate the steady state concentrations and fluxes. The concentration of extracellular Trp was set to 10 mM, corresponding to the upper bound of free Trp concentrations measured in blood (34).

### Bioluminescence Measurement in Reporter-TDO2 Mice

The mouse line was developed and previously described by Lanz et al. (35). Homozygous reporter-TDO2 were injected with 50 mg/kg d-luciferin (StayBrite™, BioVision, Mountain View, CA, USA) 5 min before imaging. The reporter mice were anaesthetized using isoflurane and assayed for bioluminescence intensity using an *in vivo* Imaging System (IVIS, Caliper/PerkinElmer, Waltham, MA, USA) with a cooled charged-coupled device (CCD) camera. Following baseline imaging prior to treatment, three mice each were injected i.p. with 8 mg/mouse DMOG (Frontier Scientific Inc.) dissolved in saline (0.9% NaCl) (Fresenius Kabi), following which a second bioluminescence imaging was performed 24 h later. Bioluminescence images were acquired as photons/s/cm^2^/steradian over a period of 60 s using the Living Image software (version 2.50, Caliper/PerkinElmer). Signal quantification was done by measuring emitted photons from acquisition regions. Bioluminescence was plotted as fold change compared to pre-treatment baseline levels. The experiments were approved under the animal grant number G240/18 by the governmental review committee on animal care of Baden-Württemberg, Germany.

### Statistical Analysis

GraphPad Prism v8.02.1 (GraphPad Software Inc.) was used for statistical analysis. For single comparisons between two data sets, unpaired two-tailed Student’s t-tests were utilized. Rank sum analysis by Mann-Whitney U test was carried out wherever necessary. All data are plotted as mean (± SEM), unless stated otherwise. Differences with a p ≤ 0.05 were considered to be statistically significant (ns non significant; p > 0.05; * p ≤ 0.05; ** p ≤ 0.01; *** p ≤ 0.001; **** p ≤ 0.0001).

## Results

### Hypoxic Exposure Downregulates Constitutive *Tdo2* Expression in Hepatocytes

**Figure 1:**
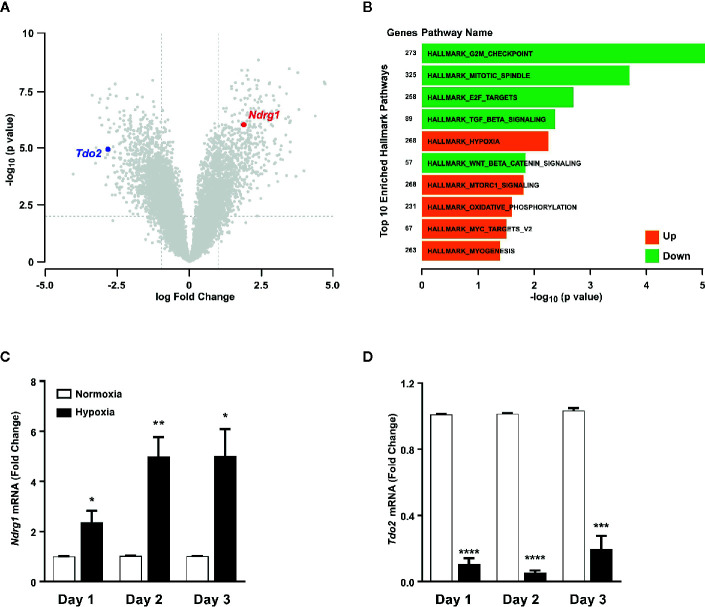
Tryptophan-2,3-dioxygenase (*Tdo2*) expression is reduced in primary murine hepatocytes exposed to hypoxia. **(A)** Volcano plot showing the differentially regulated genes in microarray data of primary murine hepatocytes after exposure to 48 h hypoxia (1% O_2_) compared to control hepatocytes exposed to 48 h normoxia (19% O_2_). (n=3) **(B)** Differentially regulated top 10 hallmark pathways enriched in the microarray data shown in **(A)**. **(C)** mRNA expression of the surrogate hypoxia marker gene *Ndrg1* measured by (q)RT-PCR in primary murine hepatocytes after 1, 2 or 3 days of exposure to either normoxia (white) or hypoxia (back). (n=3) **(D)** Expression of *Tdo2* mRNA measured by (q)RT-PCR in primary murine hepatocytes as in **(C)**. Data represented as mean ± S.E.M. Statistical significance was assumed at p < 0.05 (*p < 0.05, **p < 0.01, ***p < 0.001, ****p ≤ 0.0001).

Microarray analysis of mRNA expression in cultured primary murine hepatocytes post exposure to 48 h of hypoxia (1% O_2_), revealed a strong reduction in the expression of *Tdo2* mRNA (**Figure 1A**). Establishment of intra-cellular hypoxia was confirmed by increased expression of the surrogate hypoxia marker *Ndrg1* and also upregulation of the Hallmark_Hypoxia pathway genes (**Figure 1B**). The results of the microarray were validated by (q)RT-PCR measurements (**Figures 1C, D** **)** not only at 48 h but also across all measured time points. The measurements revealed elevated *Ndrg1* mRNA levels (**Figure 1C**) in hypoxic hepatocytes, while constitutive *Tdo2* mRNA expression was reduced across all time points (**Figure 1D**). These observations are in line with our prior observations in glioblastoma cells, indicating that downregulation of TDO2 might be a global response to hypoxia.

### Hypoxia Mediated *Tdo2* Downregulation in Hepatocytes Reduces Trp Flux

**Figure 2:**
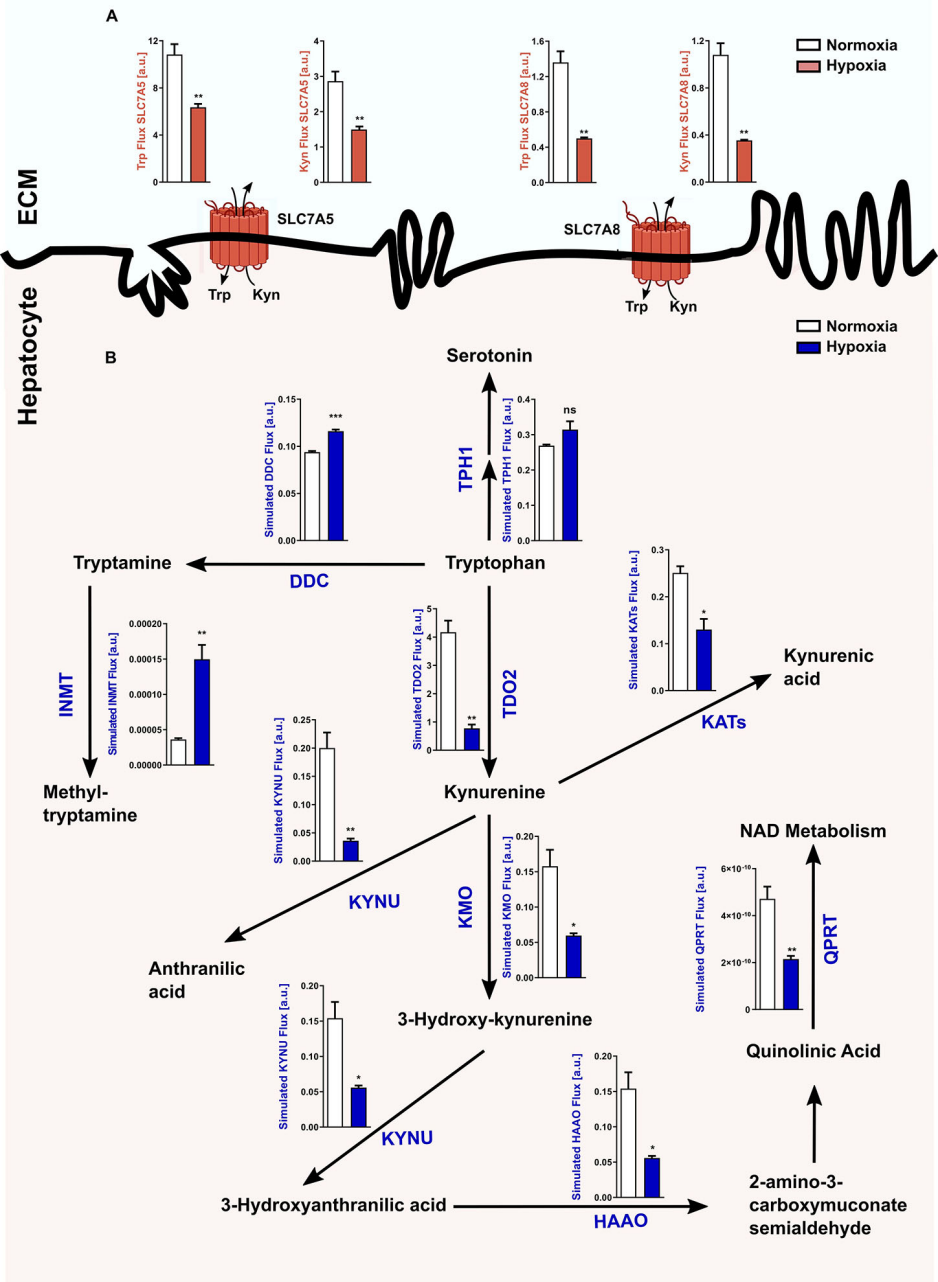
Reduced *Tdo2* expression leads to reduced Trp flux through the kynurenine pathway (KP). **(A)** Predicted flux through the large amino acid transporters SLC7A5 and SLC7A8 in hepatocytes under hypoxia (maroon bars) as compared to control normoxic hepatocytes (white bars). The abbreviations used for the transporters are SLC7A5—large neutral amino acids transporter small subunit 1 (LAT1) and SLC7A8—large neutral amino acids transporter small subunit 2 (LAT2). ECM – extracellular matrix (n=3). **(B)** Graphical depiction of the changes in the flux through the different enzymes involved in the Trp metabolic pathway upon exposure to either normoxia (white bars) or hypoxia (blue bars). Microarray data generated from primary murine hepatocytes exposed to either 48 h hypoxia or normoxia as described in **Figure 1A**, was integrated into a mathematical model of Trp metabolism to calculate the fluxes through the different enzymes. (n=3) The abbreviations used for the enzyme names are DDC: dopa decarboxylase; TPH1: L-tryptophan hydroxylase 1; IL4I1: interleukin 4 induced 1; INMT: indolethylamine methyltransferase; KATs: kynurenine aminotransferases; KYNU: kynureninase; KMO: kynurenine 3-monooxygenase; HAAO: hydroxyanthranilic acid oxidase and QPRT: quinolinic acid phosphoribosyltransferase. Data represented as mean ± S.E.M. Statistical significance is assumed at p < 0.05 (*p < 0.05, **p < 0.01, ***p < 0.001). ns, not significant.

In light of the hypoxia-mediated downregulation of *Tdo2* expression, we next conducted an *in silico* analysis to study the global effects of hypoxia on Trp metabolism in hepatocytes. Microarray-based RNA expression data of Trp pathway enzymes and transporters, together with other predefined modelling parameters were fed into a previously described Trp metabolism model (31), which shows that under hypoxic conditions a significant decrease in the Trp-Kyn exchange by the well-studied large amino acid transporters LAT1 (SLC7A5) and LAT2 (SLC7A8) is observed (**Figure 2A**). Further, hypoxia also reduced hepatic Trp flux through TDO2 into the KP, which in turn decreased the metabolic flux through downstream KP enzymes such as kynureninase (KYNU), kynurenine aminotransferases (KATs), kynurenine 3-monooxygenase (KMO) (**Figure 2B**). Enzymatic flux was also reduced under hypoxia further downstream in the KP such as through the enzymes hydroxyanthranilic acid oxidase (HAAO) and quinolinic acid phosphoribosyltransferase (QPRT) i.e. the enzymes that feed into the *de novo* NAD synthesis pathway (**Figure 2B**). In contrast, flux through the serotonin synthesis pathway catalyzed by tryptophan hydroxylase 1 (TPH1) (**Figure 2B**) showed a tendency towards an increase. Interestingly, inspite of the reduced flux of Trp into the hepatocytes under hypoxia, there was an increased flux of Trp through dopa decarboxylase (DDC), which converts Trp to tryptamine and subsequently also through indolethylamine methyltransferase (INMT), which further methylates tryptamine to methyltryptamine and dimethyltryptamine (DMT) (**Figure 2B**). These results indicate that reduced oxygen availability reduces Trp metabolism down the KP, while promoting the production of tryptamine and its downstream metabolites.

**Figure 3:**
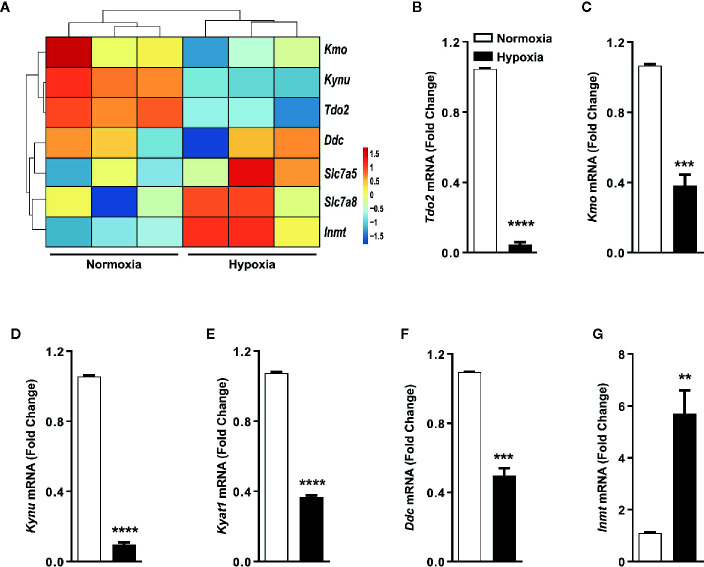
Trp transporters and other Trp pathway enzymes are differentially regulated by hypoxia. **(A)** Heatmap depicting microarray data as in **Figure 1A** used for generating the Trp metablic model, showing the differential regulation of mRNA expression of Trp pathway enzymes and Trp transporters under hypoxia. (n=3) **(B–G)** Expression of Trp pathway enzymes mRNA measured by (q)RT-PCR validating the microarray data for expression of **(B)** *Tdo2*, **(C)** *Kmo*, **(D)** *Kynu*, **(E)** *Kyat1*, **(F)** *Ddc*, **(G)** *Inmt*. (n=3) Data represented as mean ± S.E.M. Statistical significance is assumed at p < 0.05 (**p < 0.01, ***p < 0.001, ****p ≤ 0.0001).

Gene expression analysis by microarray revealed that the expression of TDO2 and other KP enzymes was reduced under hypoxic exposure, while expression of non-KP enzymes such as INMT was increased upon reduced oxygen availability (**Figure 3A**), which was confirmed by qRT-PCR analysis (**Figures 3B–G**).

### Measurement of Intra-Cellular Trp Derived Metabolites Validate the *In Silico* Analysis

**Figure 4:**
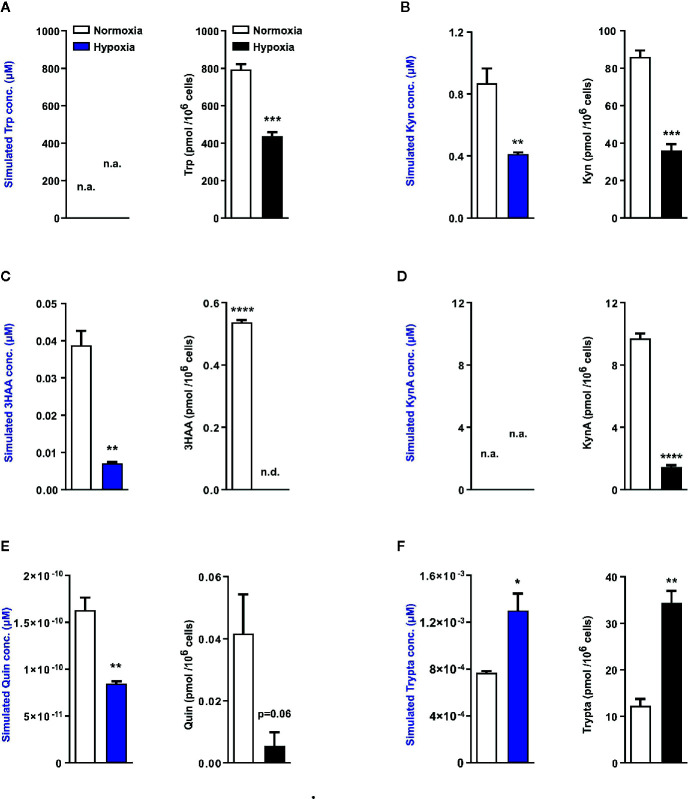
Mathematical modelling and uHPLC measurements predicts a complete rewiring of hepatic Trp catabolism under hypoxia. Using the microarray data as described in **Figure 2**, the model predicts the intracellular metabolite concentrations in primary murine hepatocytes exposed to normoxia (white bars) or hypoxia (blue bars) for 48 h (n=3). uHPLC measurements of intracellular metabolite concentrations of hepatocytes cultured for 48 h either under normoxia (white bars) or hypoxia (black bars) (n=3). These plots reveal intracellular predictions for changes in concentration and actual measured intracellular concentrations of various Trp metabolic products **(A)** tryptophan (Trp), **(B)** kynurenine (Kyn), **(C)** 3-hydroxyanthranilic acid (3HAA), **(D)** kynurenic acid (KynA), **(E)** quinolinic acid (Quin) and **(F)** tryptamine (Trypta). The external Trp concentration is kept constant and is fed as an input into the model thus predictions for Trp changes are not available, while the model lacks sufficient enzymatic parameters for KynA change predictions therefore the respective plots indicate n.a., not available. Data represented as mean ± S.E.M. Statistical significance is assumed at p < 0.05 (*p < 0.05, **p < 0.01, ***p < 0.001, ****p ≤ 0.0001). n.d. - not detected/below detection limit.

Next using the aforementioned Trp model, we carried out *in silico* predictions which depicted reduced KP metabolite concentrations downstream of TDO2 (blue plots, **Figures 4A–E**). The increased flux through DDC as observed earlier in **Figure 2B**, was also reflected in the simulated tryptamine concentration (blue plot, **Figure 4F**). These modelling results are indicative of the possibility of an extensive reprogramming of Trp metabolism in hepatocytes upon exposure to hypoxia. To verify these *in silico* predictions, we next performed metabolic analysis of the hepatocytes cultured under hypoxic conditions for 48 h, which revealed a diminished intracellular Trp pool under hypoxia (black plot, **Figure 4A**), which is in line with the predicted reduction in Trp uptake by LAT1 and LAT2 (**Figure 2A**). The intracellular concentrations of Kyn (black plot, **Figure 4B**), 3HAA (black plot, **Figure 4C**) and KynA (black plot, **Figure 4D**) were also significantly reduced in hepatocytes exposed to oxygen restricted conditions.

The quinolinic acid levels measured in hypoxic hepatocytes were also decreased (black plot, **Figure 4E**), validating the predicted reduction in quinolinic acid (blue plot, **Figure 4E**). This hypoxia induced decrease in quinolinic acid, a precursor required for *de novo* NAD synthesis, resulted in reduced abudance of NAD, NADH, NADPH as well as other energy currencies in the hepatocytes (**Figure S1A–S1E**). Of note, in line with the prediction of increased tryptamine levels under hypoxia (blue plot, **Figure 4F**), a significantly increased concentration of tryptamine was measured inside hypoxic hepatocytes (black plot, **Figure 4F**). Taken together, these metabolic measurements validate the results of the Trp metabolic model and demonstrate that in hepatocytes hypoxic exposure restricts the KP, while simultaneously channeling Trp towards tryptamine production.

### HIF Stabilization Reduces *Tdo2* Expression in Mouse Liver

**Figure 5:**
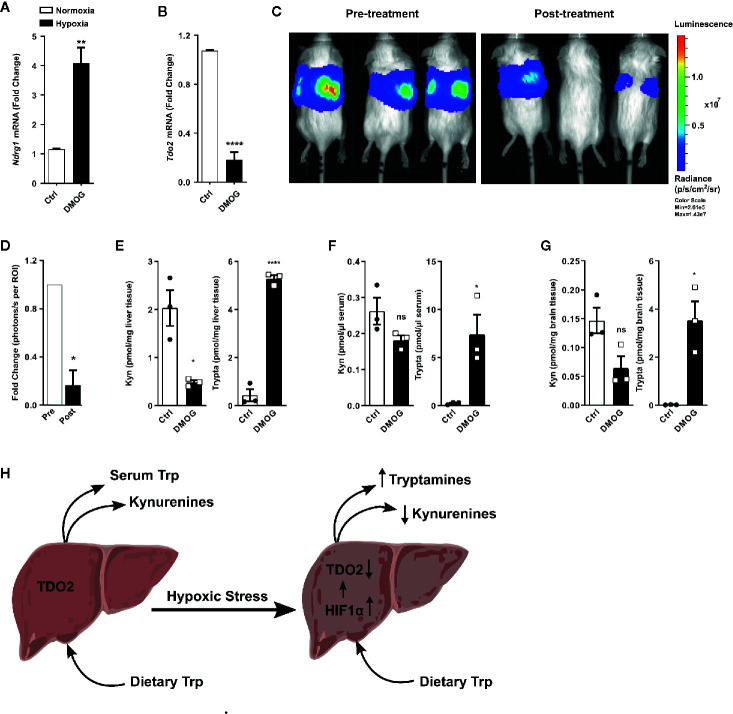
HIF stabilization suppresses hepatic *Tdo2* expression, resulting in elevated tryptamine levels in the liver, serum and brain. **(A)** mRNA expression analysis of *Ndrg1* by (q)RT-PCR in primary murine hepatocytes upon HIF stabilization by exposure to 3 mM DMOG for 24 h. (n=4) **(B)** Expression of *Tdo2* mRNA in primary murine hepatocytes after treatment with 3 mM of the HIF-stabilizing agent, DMOG for 24 h. (n=4) **(C)** IVIS image of 6 weeks old *Tdo2* reporter mice at baseline pre DMOG treatment (left) and 24 h post DMOG treatment (right). **(D)** Quantification of the bioluminescence signal measured by IVIS (n=3). **(E–G)** LC-MS/MS based measurements of kynurenine and tryptamine levels in **(E)** liver tissue, **(F)** serum and **(G)** brain tissue of WT C57BL/6 mice treated with saline control (white bars) or 8 mg/mouse DMOG (black bars) for 24 h (n=3). **(H)** Graphical summary depicting increased tryptamine and decreased kynurenine production in liver under hypoxic stress, due to reduced TDO2 expression. Data are expressed as mean ± S.E.M. Statistical significance is assumed at p < 0.05 (*p < 0.05, **p < 0.01, ****p ≤ 0.0001). ns, not significant.

A majority of the cellular effects of hypoxia are known to be mediated by stabilization of the HIF master regulators. Therefore, we next explored if HIFs also regulate the metabolic fate of Trp in hepatocytes. To this end, we employed the well-known chemical hypoxia mimetic dimethyloxalylglycine (DMOG), which acts by inhibiting the prolyl-hydroxylase enzymes and thus stabilizes the HIF proteins even under sufficient oxygen availability. Upon exposure to DMOG, cultured primary mouse hepatocytes significantly upregulated the HIF-controlled hypoxia response gene *Ndrg1* (**Figure 5A**), while simultaneously downregulating the expression of *Tdo2* (**Figure 5B**).

To explore whether the downregulation of *Tdo2* upon HIF stabilisation also occurs *in vivo*, we utilised *Tdo2* reporter mice (35). In these mice, the *Tdo2* gene sequence has been switched with a luciferase gene, which is still under the regulatory control of the *Tdo2* promoter, thus enabling the study of *Tdo2* gene expression *in vivo*. For the current study, these mice were subjected to an initial baseline bioluminescence measurement (**Figure 5C**, left) immediately before treatment with DMOG. Twenty-four hours post DMOG treatment, a second bioluminescence measurement was performed. The bioluminescence signal intensity was strongly reduced after DMOG treatment (**Figure 5C**, right) as compared to the baseline measurement. Quantification of the bioluminescence revealed a significant reduction in signal intensity in response to DMOG (**Figure 5D**), which signifies less expression of *Tdo2*.

We next investigated the HIF-induced changes in Trp homeostasis *in vivo.* Metabolic measurements revealed that after 24 h of DMOG treatment the Kyn levels in the livers of the mice were significantly reduced, while a trend towards reduced Kyn levels was observed in the serum and the brain tissue of the mice (**Figures 5E–G**). Of note, significantly elevated levels of tryptamine were measured in the liver, serum and brain of DMOG treated mice (**Figures 5E–G**). Taken together these results show that HIF stabilization *in vivo* results in an increased production of tryptamines, while simultaneously reducing KP metabolites (**Figure 5H**).

### Maintainance of Hepatic AHR Activation Under Hypoxia

Many reports associate the AHR with Trp metabolite dependent cellular effects (36). We therefore hypothesized that the reduced production of Kyn and downstream metabolites under hypoxia could potentially reduce AHR activity in the hepatocytes. To check the status of AHR activation in the hypoxic hepatocytes, we explored the expression data generated by the microarray in **Figure 1A**. The top table (**Table S2**) revealed the upregulation of many well-known AHR target genes (listed in **Table 1**). Furthermore, bioinformatics analysis of the data also revealed genes in the Reactome_Xenobiotics pathway to be one of the top 10 upregulated curated pathways in hypoxic hepatocytes (**Figure S2A**).

**Table 1:** Top known AHR target genes, whose expression was upregulated under hypoxia.

No.	AHR target gene	Fold Change	Average Expression	p-value	Adjusted p-value	Ref.
1	*Cyp2e1*	11.179	8.725	2.86E-09	1.24E-05	(37)
2	*Cyp4a12a*	10.241	7.111	2.48E-06	0.000165	(37)
3	*Cyp2s1*	7.771	6.703	4.73E-05	0.000889	(38)
4	*Cyp1a2*	6.555	8.567	3.88E-07	6.82E-05	(37)
5	*Cyp17a1*	5.644	6.373	6.88E-05	0.001092	(39)
6	*Cyp3a11*	5.348	8.928	2.37E-05	0.000577	(40)
7	*Areg*	5.314	6.434	1.56E-05	0.000461	(41)
8	*Cyp2c54*	5.174	6.974	0.000108	0.00144	(42)
9	*Cyp2f2*	5.095	9.577	2.23E-08	1.48E-05	(38)
10	*Ces2a*	4.372	8.393	2.41E-07	5.03E-05	(42)
11	*Cyp51*	4.264	8.083	8.50E-07	9.98E-05	(43)
12	*Cyp3a16*	4.218	8.578	5.78E-05	0.001001	(38)
13	*Cyp3a41b*	3.110	7.323	0.000117	0.001537	(44)
14	*Tiparp*	2.587	7.120	0.000166	0.00189	(45)
15	*Gsn*	2.576	7.330	5.71E-05	0.000993	(46)
16	*Aldh3a1*	2.221	7.100	0.001105	0.00648	(38)

The microarray data shown in **Figure 1A** was used for this analysis. For generating this table a fold change cut-off of 2 was considered. The fold change, average expression, p values and the references showing that the genes are AHR target genes are indicated. The data was generated from at least three independent experiments using primary murine hepatocytes cultured in vitro for 48 h exposed to hypoxia (1% O_2_) and compared to control primary murine hepatocytes cultured under 48 h normoxia (19% O_2_) conditions. For the complete list of all upregulated genes with a fold change ≥ 2 refer to **Table S2**.

Previous studies reported tryptamine-mediated AHR activation (47). We therefore hypothesized that the observed AHR activity under hypoxia could be attributed to the increased tryptamine levels. To test this hypothesis, we treated murine hepatocytes with tryptamine. Tryptamine treatment induced the mRNA expression levels of the two well-known AHR target genes *Areg* and *Tiparp* (**Figures S2B, S2C**), suggesting that tryptamine increases AHR activity upon accumulation. Taken together, these observations are indicative of maintainance of AHR-mediated transcriptional activity in hypoxic hepatocytes even upon reduced KP metabolite production.

## Discussion

In the human body, the liver is at the very core of a range of systemically important processes such as digestion, nutrient absorption, detoxification, immunity, as well as the regulation of systemic nutrient availability and metabolism (1, 3, 48). Trp catabolism represents one such systemically important process, in which the liver plays a central role (4). Many ailments of the liver as well as circulatory and pulmonary systems can adversely impact hepatic blood flow and oxygen availability, thus resulting in portal hypoxemia and subsequent hepatic hypoxia (49, 50). Occurrence of hepatic hypoxia can impair hepatic functionality and thus adversely affect systemic metabolic homeostasis. Recent reports have highlighted hypoxia-mediated changes in the expression of hepatic glucose and lipid metabolic pathway genes resulting in altered liver metabolism (3, 51, 52).

In light of the aforementioned reports and given the importance of the liver in systemic Trp metabolism, we explored the effects of hypoxia on hepatic Trp homeostasis. Our results indicated that under hypoxia, murine hepatocytes downregulated the expression of *Tdo2* (**Figure 1**). Microarray analysis and qRT-PCR analyses also revealed differential expression of different Trp pathway transporters and enzymes under exposure to oxygen limiting conditions (**Figure 3**). The discovery of this regulatory effect aroused our curiosity regarding the effect of hypoxia on the production of different Trp metabolites both dependent as well as independent of TDO2. Modelling of Trp metabolism as well as metabolic measurements showed that hypoxia significantly reduced the flow of Trp into the KP, thus resulting in the reduced production of KP metabolites such as Kyn, KynA, 3HAA and QA (**Figures 2** and **4**), many of whom play a role in systemically important processes such as immune modulation, neuro-transmission and are also precursor feeder molecules into other metabolic pathways such as the *de-novo* NAD synthesis pathway. Furthermore, our experiments also revealed the simultaneous channeling of Trp metabolism towards non-KP pathways under hypoxia. In line, increased Trp flux through the non-KP enzyme DDC (**Figure 2B**) led to increased production of the Trp metabolite tryptamine (**Figure 4F**) in liver hepatocytes. Previous reports of elevated tryptamine levels in patients with hypoxic liver cirrhosis (53) and in rat models of chronic ischemic tissue hypoxia (54) are consistent with our findings. Taken together, the previous reports and our current findings, establish the presence of extensively remodelled Trp metabolic pathways in the liver under pathological as well as induced hypoxic conditions, which favours increased production of tryptamine.

Tryptamine is a trace amine and it lends its name to a broad class of serotonergic hallucinogens or psychedelic compounds, long known to induce episodes of psychosis, hallucinations or altered states of consciousness (55, 56). Tryptamine and its downstream hallucinogenic metabolites including DMT are found endogenously in mammalian brain although in trace amounts (57, 58). A two step methylation of tryptamine, catalysed by indolethylamine methyltransferase (INMT), first gives rise to methyl-tryptamine and subsequently DMT (59). Our *in silico* data showed a significantly increased flux through INMT under hypoxic conditions (**Figure 2B**), which could potentially lead to increased methyl-tryptamine and DMT levels. Numerous reports indicate the occurrence of altered states of consciousness among non-acclimatized high altitude climbers and hikers (47, 60–63). Apart from the reduced oxygen availability to the brain, our current results (**Figures 4F** and **5E–G**) suggest that such experiences could be caused by increased levels of endogenous psychedelic compounds such as tryptamine and DMT. Furthermore, abnormal behavioural patterns are also common in hepatic encephalopathy, where prior studies have reported enhanced levels of endogenous tryptamines (53, 64–67), which could now be attributed to the widespread prevalence of hypoxia in the diseased liver. In addition, increased levels of DMT can potentially constitute a feedback protection mechanism against hypoxic injury via the Sigma-1 receptor, through decreased expression of the master hypoxic transcription regulator HIF protein (58).

The liver plays an important role in systemic immune tolerance, as its specially configured microenvironment hosts a repertoire of antigen presenting cells (APCs), which promotes tolerance against a range of harmless antigens often found in the diet (68, 69). These resident APCs enforce immunosuppression by production of anti-inflammatory cytokines and expression of programmed cell death ligand 1 (PD-L1) (70). Furthermore, hepatic resident APCs such as Kupffer cells are also known to employ the immunosuppressive effects of Trp catabolism in response to an inflammatory stimulus (71). The immunosuppressive effects of Trp catabolism can be mediated by a two-pronged strategy, first by depletion of Trp, as Trp is essential for the proliferation and activity of liver-infiltrating T cells (72, 73) and secondly by the AHR-mediated immunosuppressive action of the KP metabolites, as has frequently been described in extra-hepatic systems such as the placenta (74) and different types of cancers (36, 75, 76).

Mindful of the role of AHR in immune tolerance, the observed hypoxic suppression of KP metabolite production could potentially affect hepatic AHR activation and thus hamper the immunological balance of the hepatic microenvironment. However, to our surprise, we found several well-known AHR target genes as well as the Reactome_Xenobiotics pathway upregulated in hypoxic hepatocytes (**Tables 1**, **S2** and **Figure S2A**). Our experimental results revealed that accumulation of tryptamine can potentially maintain AHR activity (**Figures S2B, S2C**). Apart from the previously discussed psychedelic effects of tryptamine, both endogenous tryptamine as well as tryptamine of gut microbial origin have been reported to posses immune-modulatory effects mediated *via* AHR activation (47, 60–63). Together, previous reports as well as data from the current study clearly indicate towards the effective maintainance of essential immunosuppresive AHR activation in hepatic cells exposed to hypoxia, which could be attributed to increased production and accumulation of other non-KP Trp metabolites such as tryptamine.

In summary, our results demonstrate the conservation of HIF-mediated suppression of Trp catabolism along the KP both in malignant as well as primary cells constitutively expressing TDO2, which illustrates the significance of this mechanism not only in pathology but also in normal physiology. In the liver cells, this suppression leads to the complete re-wiring of the Trp metabolic pathways favouring the tryptamine branch. However, the increase in tryptamine generated through DDC, the only Trp-metabolizing enzyme which does not require oxygen, appears to be specific for hepatocytes as an increase in tryptamine was not observed in hypoxic glioblastoma cells (21). This channeling of Trp into tryptamine and its downstream metabolites methyl-tryptamine and DMT could explain the “out of the body” experiences reported under oxygen-limited conditions. Furthermore, the hypoxic upregulation of the tryptamine branch of Trp metabolism, could potentially be the reason for the maintainance of immunosuppresive AHR activation despite the suppression of the KP branch of Trp catabolism.

## Data Availability Statement

The datasets generated during this study are available publicly in the Gene Expression Omnibus 429 (GEO) repository under accession number GSE159320.

## Ethics Statement

The animal study was reviewed and approved by Regierungspräsidium, Karlsruhe, Germany.

## Author Contributions

SM and CO designed the study. SM, GP, and CO developed methodology. PL and UK isolated primary murine hepatocytes. TL and MP developed and provided *Tdo2* reporter mice. SM, HG, and GP acquired data. SM, AS, SS, GP, HG, IH, and CO analyzed and interpreted data. SM and CO wrote the manuscript. All authors contributed to the article and approved the submitted version.

## Funding

This work was supported by grants from the BMBF e:Med initiative (GlioPATH, 01ZX1402), the European Union’s Horizon 2020 research and innovation programme under Grant 754688 (MESI-STRAT), DFG grant 406052676, PL-315/5-1 and the Liver Systems Medicine network (LiSyM, 031L0042).

## Conflict of Interest

The authors declare that the research was conducted in the absence of any commercial or financial relationships that could be construed as a potential conflict of interest.

## Supplementary Material

Supplementary Figure 1 Hypoxia downregulates cellular energetics in cultured murine hepatocytes. LC-MS/MS measurements of intracellular energy currencies in hepatocytes cultured for 48 h either under normoxia (white bars) or hypoxia (black bars) (n=4). These plots reveal intracellular concentrations of **(A)** nicotinamide adenine dinucleotide (NAD), **(B)** reduced NAD (NADH), **(C)** reduced nicotinamide adenine dinucleotide phosphate (NADPH), **(D)** adenosine diphosphate (ADP) and **(E)** adenosine triphosphate (ATP) Data represented as mean ± S.E.M. Statistical significance is assumed at p < 0.05 (*p < 0.05, **p < 0.01, ***p <0.001, ****p ≤ 0.0001). n.d. - not detected/below detection limit.

Supplementary Figure 2
**(A)** Top 20 differentially enriched curated pathways, with top 10 up-regulated (orange) and top 10 down-regulated (green) pathways in the microarray data shown in **Figure 1A**. The data was generated from at least three independent experiments using primary murine hepatocytes after exposure to 48 h hypoxia (1% O_2_) compared to control hepatocytes exposed to 48 h normoxia (19% O_2_). **(B)** Analysis of AHR activation upon treatment of murine hepatocytes with tryptamine by (q)RT-PCR based mRNA expression analysis of the AHR target gene *Areg* (n=3) **(C)**. AHR activity test as described in B by (q)RT-PCR based mRNA expression analysis of the AHR target gene *Tiparp* (n=3) Data are expressed as mean ± S.E.M. Statistical significance is assumed at p < 0.05 (*p < 0.05, **p < 0.01, ****p ≤ 0.0001).

Supplementary Table 2 Complete list of all upregulated genes in hypoxic primary murine hepatocytes with a fold change ≥ 2.
