# Supplementary figures and images for: Hypoxia Routes Tryptophan Homeostasis Towards Increased Tryptamine Production
Source: Front Immunol. 2021 Feb 19;12:590532. doi: 10.3389/fimmu.2021.590532 (PMC7933006; doi:10.3389/fimmu.2021.590532)

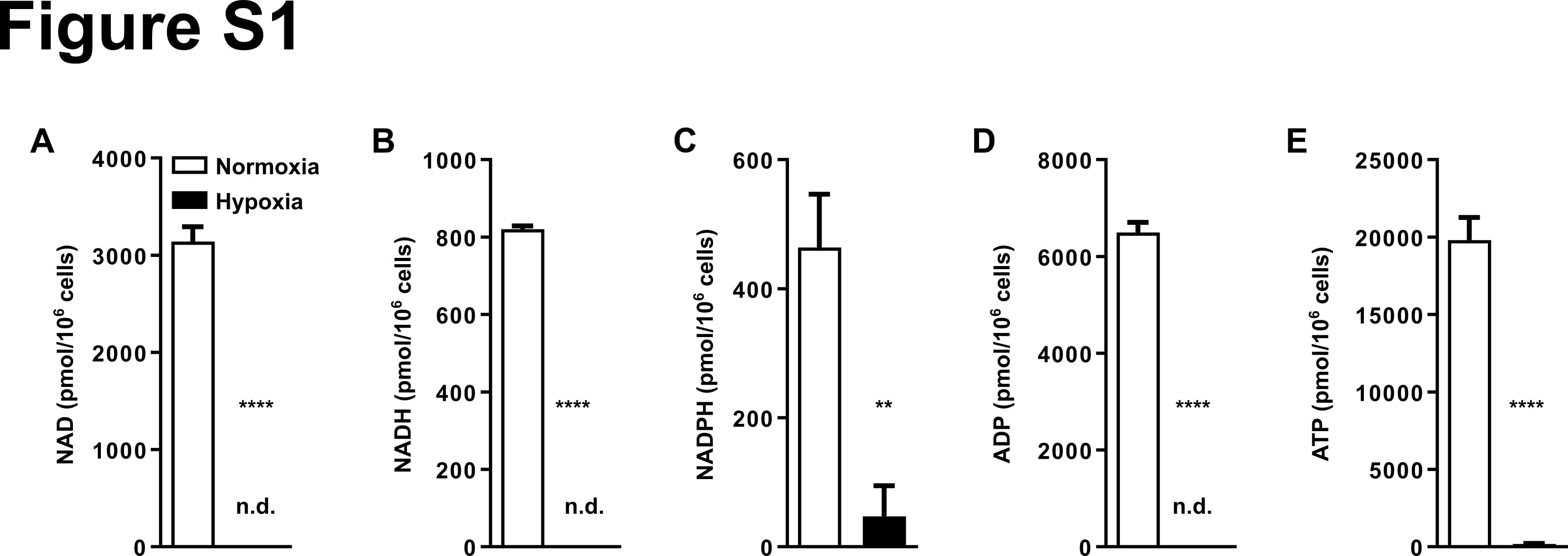

Supplement: Supplementary Figure 1 — Hypoxia downregulates cellular energetics in cultured murine hepatocytes. LC-MS/MS measurements of intracellular energy currencies in hepatocytes cultured for 48 h either under normoxia (white bars) or hypoxia (black bars) (n=4). These plots reveal intracellular concentrations of (A) nicotinamide adenine dinucleotide (NAD), (B) reduced NAD (NADH), (C) reduced nicotinamide adenine dinucleotide phosphate (NADPH), (D) adenosine diphosphate (ADP) and (E) adenosine triphosphate (ATP) Data represented as mean ± S.E.M. Statistical significance is assumed at p < 0.05 (*p < 0.05, **p < 0.01, ***p <0.001, ****p ≤ 0.0001). n.d. - not detected/below detection limit. [file Image_1.jpeg]

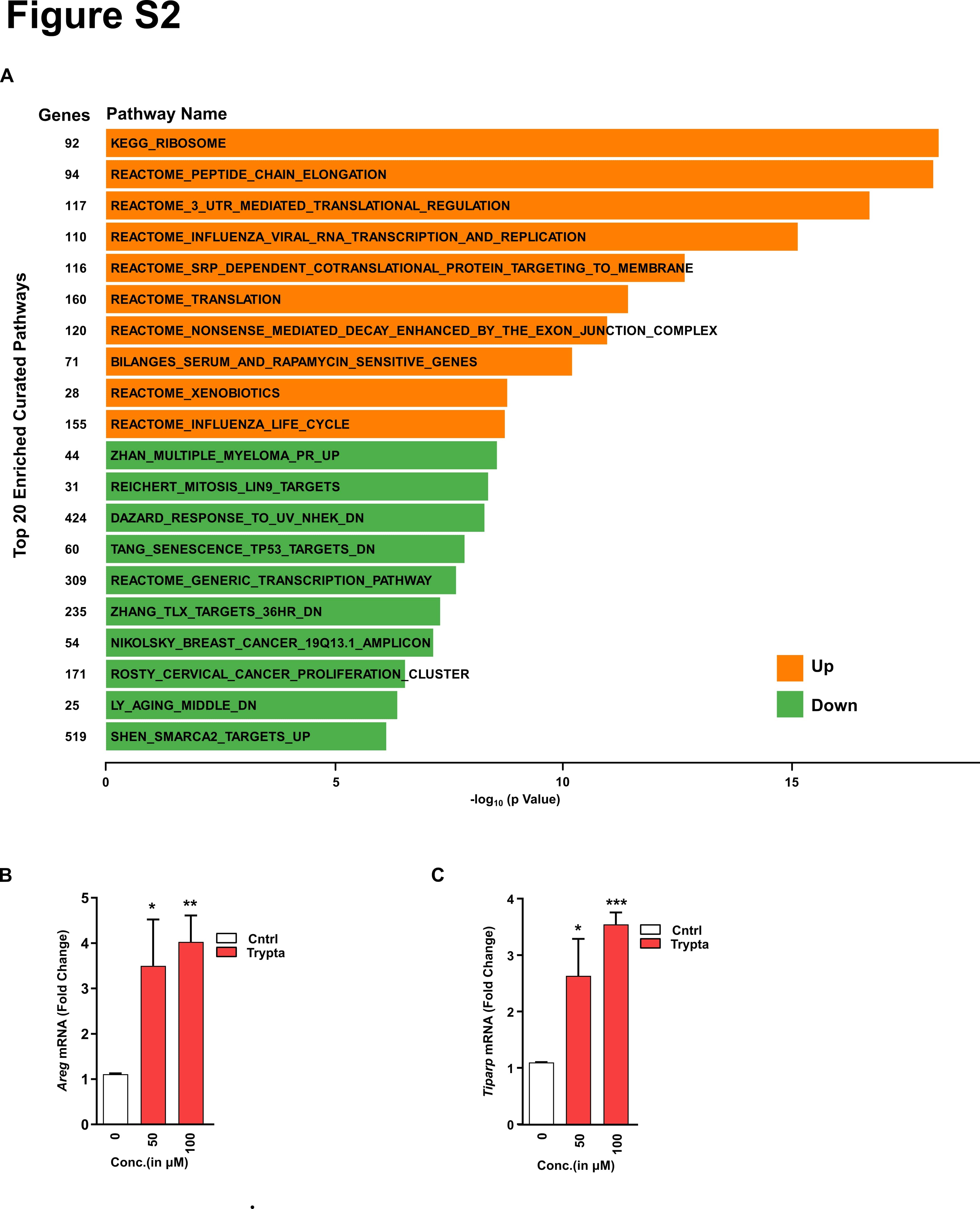

Supplement: Supplementary Figure 2 — (A) Top 20 differentially enriched curated pathways, with top 10 up-regulated (orange) and top 10 down-regulated (green) pathways in the microarray data shown in Figure 1A . The data was generated from at least three independent experiments using primary murine hepatocytes after exposure to 48 h hypoxia (1% O2) compared to control hepatocytes exposed to 48 h normoxia (19% O2). (B) Analysis of AHR activation upon treatment of murine hepatocytes with tryptamine by (q)RT-PCR based mRNA expression analysis of the AHR target gene Areg (n=3) (C). AHR activity test as described in B by (q)RT-PCR based mRNA expression analysis of the AHR target gene Tiparp (n=3) Data are expressed as mean ± S.E.M. Statistical significance is assumed at p < 0.05 (*p < 0.05, **p < 0.01, ****p ≤ 0.0001). [file Image_2.jpeg]
